# Land Use Dynamics of the Fast-Growing Shanghai Metropolis, China (1979–2008) and its Implications for Land Use and Urban Planning Policy

**DOI:** 10.3390/s110201794

**Published:** 2011-01-31

**Authors:** Hao Zhang, Li-Guo Zhou, Ming-Nan Chen, Wei-Chun Ma

**Affiliations:** Department of Environmental Science and Engineering, Fudan University, 220 Handan road, Shanghai 200433, China; E-Mails: zhokzhok@163.com (H.Z.); zhouli-guo@tom.com (L.G.Z.); 09210740001@fudan.edu.cn (M.N.C.)

**Keywords:** land use and land cover change (LULC), land use dynamics, remote sensing, GIS, Shanghai, China

## Abstract

Through the integrated approach of remote sensing and geographic information system (GIS) techniques, four Landsat TM/ETM+ imagery acquired during 1979 and 2008 were used to quantitatively characterize the patterns of land use and land cover change (LULC) and urban sprawl in the fast-growing Shanghai Metropolis, China. Results showed that, the urban/built-up area grew on average by 4,242.06 ha yr^−1^. Bare land grew by 1,594.66 ha yr^−1^ on average. In contrast, cropland decreased by 3,286.26 ha yr^−1^ on average, followed by forest and shrub, water, and tidal land, which decreased by 1,331.33 ha yr^−1^, 903.43 ha yr^−1^, and 315.72 ha yr^−1^ on average, respectively. As a result, during 1979 and 2008 approximately 83.83% of the newly urban/built-up land was converted from cropland (67.35%), forest and shrub (9.12%), water (4.80%), and tidal land (2.19%). Another significant change was the continuous increase in regular residents, which played a very important role in contributing to local population growth and increase in urban/built-up land. This can be explained with this city’s huge demand for investment and qualified labor since the latest industrial transformation. Moreover, with a decrease in cropland, the proportion of population engaged in farming decreased 13.84%. Therefore, significant socio-economic transformation occurred, and this would lead to new demand for land resources. However, due to very scarce land resources and overload of population in Shanghai, the drive to achieve economic goals at the loss of cropland, water, and the other lands is not sustainable. Future urban planning policy aiming at ensuring a win-win balance between sustainable land use and economic growth is urgently needed.

## 1. Introduction

Land use and land cover change (LULC) and the subsequent impacts on the planet have been one of the increasing focus of on-going global environmental change studies. Among the driving forces of global environmental change, human-induced LULC has been recently recognized as one of the major factors in shaping local and regional ecological processes, as well as affecting ecosystem functioning [1–5]. It is argued that human transformations of ecosystems and landscapes such as appropriating and altering the Earth’s resources for human needs should be responsible for rapid changes in biogeochemical cycles and hydrologic processes, which affect provision of ecosystem services and hence human well-being [6]. Urbanization, to a large degree, represents human objectives of improving life standard and therefore drives LULC. With the ongoing urbanization a vast population is inclined to concentrate in urban areas. It is estimated that more than 50% of the world population lives in the urban areas, and it is projected that the urban population proportion will reach 69.6% by 2050 [7]. Globally, rapid urbanization and population growth have been a common phenomenon, especially in the developing countries with an increasing desire for prosperity [8]. Nowadays, 70% of the world’s largest cities are found in the developing world [9].

As the largest developing country in the world, China has experienced prosperous economic growth since 1978. Over the past decades the use of huge amounts of cropland, forest, grassland, water bodies has been changed to meet the strong demand for urban and industrial development. Socially and economically, as happened in developed and the other developing countries, such a great change led to accelerated urban expansion and drove more people, especially who lost their land do to the changes, to seek employment opportunities and settle in urban areas. Nowadays many big cities of the coastal economic zone, especially the megacities (such as Beijing, Tianjin, Nanjing, Shanghai, and Guangzhou, *etc*.) on the rim of the Bohai Sea, the Yangtze River basin, and the Pearl River delta, have been the locomotives propelling China’s economic growth [10]. Thus these big cities are the preferred destination for millions of internal migrants and overseas investors. However, China has faced a bottleneck during its rapid development due to land scarcity, population pressure, and environmental degradation. This negative situation has been more predominant in the coastal big cities. They have attracted much attention, not only due to their economic power and productivity, but also for their problems in meeting the challenge of sustainability. To a great extent, acquiring accurate and timely information on the past history, present status, and trends of human-dominated ecosystem has attracted researchers and policy decision makers [11]. However, due to the lack of timely information and overall knowledge presenting land use dynamics and driving forces, it is still difficult to quantify LULC and associated adverse effects on the environment during rapid urbanization periods. Recently, with the widely used integrated approach of using multi-temporal remote sensing and geographical information systems (GIS), this quantification has been possible. Given the importance of urban growth, LULC and their long-term adverse effects on ecological functioning, modeling LULC and urban growth has been the focus of much research [12–15]. However, less attention has been given in the development of these models to understanding the relationships between urban growth and related socioeconomic process that underlies land use change and urbanization [16].

The present study focuses on the Shanghai Metropolis, which is the most populous city in China. It was well known as the biggest financial center and an important international city in the Far East during 1920s–1930s, with thriving global and local trade [17–18]. Unfortunately, this city suffered a long period of recession during 1950s and early 1980s due to the ossified political and planned economic systems. During this period its role was changed from the financial center to China’s most important industrial city, a change that negatively impacted Shanghai’s economy. Since the national strategy of ‘opening Pudong to the world’ initialized in 1990, this city has experienced an unprecedented process of rapid urbanization. Due to its location advantages, economic power, and international fame, this representative area lends itself well for providing a useful example. Therefore, the purpose of this study was to quantitatively characterize the LULC and urban sprawl patterns by using remote sensing and GIS techniques. We believe this will lead to a better understanding of spatio-temporal patterns of land use and urban expansion, which will meet the practical needs of urban planning and environmental management, to shape rational urban clusters, and relieve the adverse effects of the human-dominated ecosystem.

## 2. Study Area

**Figure 1.**
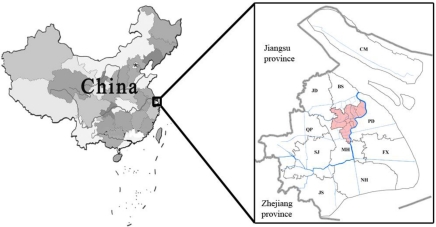
Location of the Shanghai metraopolis. Note: the highlighted center part in this figure is the city proper of Shanghai

The study area is the Shanghai Metropolis. It is located between latitudes 31°32′N and 31°27′N, and longitudes 120°52′E and 121°45′E (Figure 1). This area has a northern subtropical monsoon climate, with an average annual temperature about 15 °C. High temperatures average 28 °C in the summer and 4 °C in the winter. The average annual precipitation is approximately 1,000–1,200 mm, with about 60% of rainfall being usually received during May and September. Topographically, the area is mainly located at an alluvial terrace, which is known as the Yangtze River basin. Elevation of the area ranges between 1 and 103.4 m, with an average of 4 m.

The present administrative boundaries of the Shanghai Metropolis consist of the city proper (including Hupu district/HP, Luwan district/LW, Changning district/CN, Xuhui district/XH, Putuo district/PT, Zhabei district/ZB, Jing’an district/JA, Hongkou district/HK, and Yangpu district/YP), suburban districts (including Minghang/MH, Pudong new area/PD, Jiading/JD, Baoshan/BS, Songjiang/SJ, Qingpu/QP, Nanhui/NH, Fengxian/FX, and Jingshan/JS), and Chongming county (CM). Therefore, the Shanghai Metropolis covers an area of 6,450 km^2^, with a total of 18.18 million residents [19]. Although this area accounts for only 0.05% of China’s territory, it plays a very important role in this country’s economy. In 2008, this area contributed 4.6% to this country’s total GDP. Local GDP per capita was 72,553 RMB Yuan (equivalent 10592 US dollars, current exchange rate is 6.85), approximately 3.2-fold of that nationwide average [20].

## 3. Remotely Sensed Imagery Pre-Processing, Interpretation, and Land Use Detection

### 3.1. Satellite Data and Pre-Processing

Four remotely sensed images dated August 4, 1979, May 18, 1987, April 11, 1997, and March 24, 2008, were selected for this study. All of the images were clear and nearly free of clouds. Accordingly, the study period covered about 29 years. All the images were rectified and georeferenced to the UTM map projection prior to interpretation. Subsequently, the images were resampled to 30 meters using the nearest neighbor algorithm to keep the unchanged original brightness values of pixels, and the RMSE were both found within 1 pixel. The image processing and data manipulation were conducted using algorithms supplied with the GEOSTAR 3.0® image processing software. Furthermore, ESRI ARCGIS 9.3® was used for spatial analyses.

Land use and land cover patterns during 1987 and 2004 were mapped by the use of Landsat TM and Landsat ETM+ data respectively. As part of our routine work, a field survey of land use in Shanghai has been carried out every two years since 1996. According to a predetermined classification scheme of six categories of land covers present within the study area and their image characteristics, false-color images were produced by combining bands 5, 4, and 3 of the Landsat TM/ ETM+ images. These covers include urban or built-up land, cropland, forest and shrub, water (mainly rivers, channels, and ponds), tidal land, and bare land. Herein, the classification scheme of the study area was modified on the basis of the land use classification system by China National Committee of Agricultural Divisions [21]. Subsequently, the supervised signature extraction with the maximum likelihood algorithm was employed to classify the satellite images.

### 3.2. Accuracy Assessment

Together with land use survey data derived from historical aerial photos, five 1:250,000 digitalized land use maps acquired in 1978, 1988, 1996, 2000, and 2003 as well as one SPOT image acquired in 2007 were used as the reference data for accuracy assessment of classification of Landsat TM/ ETM+ data. For each image 210 training sites were chosen to ensure that all spectral classes covering each land use and land cover category were adequately represented in the training statistics. Furthermore, the supervised signature extraction with the maxi-mum likelihood algorithm was employed to perform the classification of the satellite images. After classification, for each image 250 samples were randomly selected to check the accuracy of the classified maps. As shown in Tables 1 to 4, overall accuracy of the land use and land cover maps during 1979 and 2008 ranges between 76.4% and 82.4%. The Kappa indexes were determined to be ranging between 0.72 and 0.79, which meet the recommended value by Jassen *et al* [22], therefore these data was available for further study.

### 3.3. Land Use and Land Cover Change Detection

A cross-tabulation detection method was employed to perform land use and land cover change detection. The land use change matrix was produced, which showed quantitative data of the overall land use and cover changes between 1979 and 2008 in the study area. Based on the main types of gains and losses in each category shown by the change matrix, land use transfer images and land use transfer matrix for each category were also produced.

Besides, in order to determine the change rate of land use categories over the study periods, the single land use dynamic degree (*LUDD_single_*) and synthetic land use dynamic degree (*LUDD_synthetic_*) [23] were used. *LU_single_* and *LU_synthetic_* were computed as follows:

(1) $${\mathrm{LUDD}}_{\mathrm{single}}=\frac{\left(U_{b}-U_{a}\right)}{U_{a}\times T}\times 100\%$$

where *U_b_* and *U_a_* are the area of the land use type in time *b* and *a* respectively, *T* is the interval between *b* and *a*:

(2) $$\mathrm{LUD}D_{\mathrm{synthetic}}=\left(\frac{\sum_{i=1}^{n}\left|\Delta LU_{\mathrm{in}-i}-\Delta LU_{\mathrm{out}-i}\right|}{2\sum_{i=1}^{n}LU_{i}}\right)\times \frac{1}{T}\times 100\%\;\;\;\;(j=1,2,3......n)$$

where *LU_i_* is the area of land use class *i* in time *a*, Δ *LU_in-i_* is the total area converted from other class *j* to class *i*, and Δ *LU_out-i_* is the total area converted from class *i* to other class *j.*

## 4. Results and Discussion

### 4.1. Synoptic Analysis of LULC and Changing Trends

**Figure 2.**
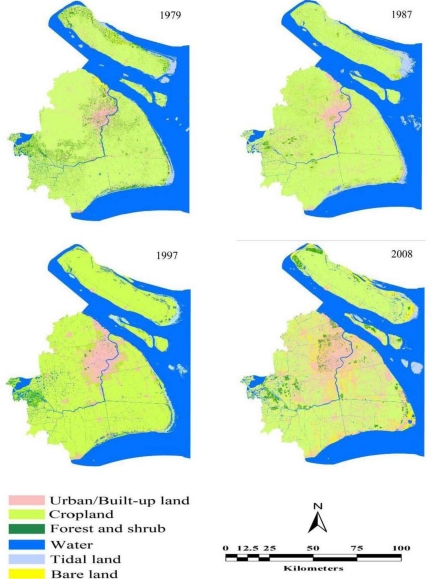
Land use maps of the study area in 1979–2008.

In general, Figure 2, Tables 5, 6, 7, and 8 show spatiotemporal patterns of land use dynamics and land cover change of the Shanghai Metropolis in 1979–2008. According to the classification results, it can be seen that extent of the land cover types varied significantly in different years. A summary general description of LULC of the Shanghai Metropolis over the study period is shown in Table 8. The urban/built-up area grew by 1,230 km^2^ between 1979 and 2008, or nearly 4,242.06 ha yr^−1^ on average. Bare land grew by 461.7 km^2^ or nearly by 1,594.66 ha yr^−1^ on average. In contrast, cropland decreased by 953 km^2^ or nearly by 3,286.26 ha yr^−1^ on average, followed by forest and shrub, water, and tidal land, which decreased on average by 1,331.33 ha yr^−1^, 903.43 ha yr^−1^, and 315.72 ha yr^−1^, respectively. It was observed that considerable former built-up land in remote rural areas was converted to other LULC types such as cropland, forest and shrub due to land reclamation associated with rural-urban migration. However, significant increase in urbanized land from the other land cover types exceeded the conversion from the urban land to the other land cover types.

**Figure 3.**
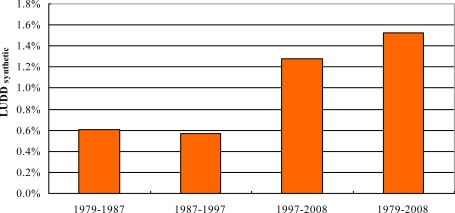
The synthetic land use dynamic degree of study area in 1979–2008.

Table 9 shows that area changes in land classes varied remarkably during each study period. Cropland, forest and shrub, water, and tidal land decreased at a low speed, whereas urban/built-up land and bare land increased at a relatively high speed. For the urban/built-up area, the change trend accelerated continuously during almost all periods, reaching the highest rate of 8.67% between 1997 and 2008 and an average dynamic rate of 7.25% between 1979 and 2008. Similarly, for the bare land, the change trend accelerated continuously except the period 1987–1997, reaching the average dynamic rate of 56.93% between 1979 and 2008. Moreover, Figure 3 shows a continuous increase trend of the land use change rate, ranging between 0.57–1.28%, with an average of 1.53% during 1979 and 2008. The rate of change of the land use in the study area has accelerated between the different time periods. Therefore, as shown in Figure 2, Tables 5, 6, 7, and 8, urban encroachment into natural and semi-natural ecosystems was detected. The result showed that approximately 83.83% of the newly urban/built-up area was converted from forest and shrub, cropland, water, and tidal land. Another significant change was the continuous incline in bare land, which was mainly caused by mining pits, stock dumps, enclosed tidal land, and abandoned croplands enclosed for development under the pressure of rapid urbanization.

### 4.2. Spatial Expansion of Urban/Built-up Lands

Figure 2, Tables 5, 6, 7, and 8 show significant land use conversion among different land use types occurred in 1979–2008. Herein, in order to well describe tempo-spatial pattern of LULC in the study area, in combination with socio-economic characteristics in different period of Shanghai, the span over the study period was divided into three stages:

Stage I (1979–1987) was a slow development period for the city. Urban/built up lands of the study area increased by 113.6 km^2^ or by nearly 1,419.85 ha yr^−1^ on average. Topographically, the Huangpu River limited spatial expansion of the city proper. Besides, urban regeneration was slowed down due to planned economic constraints. Thus, most of the newly emerging urban/built-up lands were located in the city proper, and a single-core pattern for development of the Shanghai Metropolis was apparent.

Stage II (1987–1997) was a rapid development period for urban regeneration and expansion starting in 1990, during which this city’s extent rapidly expanded to the Pudong new area due to vast foreign direct investment. As observed, urban/built-up lands of the study area increased by 230.4 km^2^ or by nearly 2,303.54 ha yr^−1^ on average. In this period, the Waigaogiao free tax zone (WFTZ), Jinqiao export Processing Zone (JEPZ), Lujiazui Finance and Trade Zone (LFTZ), Zhangjiang Hi-tech industrial park (ZHIP), and Huamu governmental administrative zone (HGAZ) were rapidly developed in the Pudong new area. This led to accelerated development of satellite towns and rural areas in the neighborhood of these newly emerging unban function zones. In contrast, spatial growth of urban/built-up lands in the other suburban districts was relatively lower.

In stage III (1997–2008) the expansion of the urban/built-up land of the Shanghai Metropolis became even faster. Urban/built-up lands of the study area increased by 886.4 km^2^ or by nearly 8,056.87 ha yr^−1^ on average. During this period four sub-civic centers of the city proper, which were known as Xujiahui, Zhenru, Wujiaochang, and Huamu [24], were developed to balance the conflicts between land use and environmental degradation associated with the single-core pattern of this city. In addition, large-scale investments were placed in the urban fringe area, especially in Pudong, Minhang, Baoshan, Songjiang, and Jiading. This resulted in the establishment of intensive industrial parks, settlements, college parks, commercial facilities, and expressway systems linking the city proper, suburban, and rural areas. As observed, both urban and satellite towns in suburban area expanded their extents remarkably. Spatially, a close linkage between the city proper, suburban area, and intensive industrial parks was established. Thus, the multi-nuclei pattern for development of the Shanghai Metropolis came into being.

### 4.3. Driving Forces Analysis of LULC

As shown in Table 8, during 1979 and 2008 the urban/built-up land increased by 210.18%, from 585.5 km2 to 1,815.5 km2. Cropland decreased by 18.9%, from 5,039.5 km2 to 4,087.1 km2. Total population has grown 66.05%, from 11.37 million to 18.88 million over the study period. Obviously, the rate of urban expansion is much greater than that of population growth. In fact, according to China’s population management policy, the total population in Shanghai and other cities consists of local registered permanent residents and regular residents from the other provinces. During the study period amount of registered permanent residents increased by 22.88%, from 11.32 million in 1979 to 13.91 million in 2008. In contrast, the amount of regular residents increased by a factor of 98.40, from 0.05 million in 1979 to 4.97 million in 2008. The increase rate of regular residents was nearly 45.82 times higher than the increase in urban/built-up land. Thus, compared with low growth in registered permanent residents, a continuous increase in regular residents played very important role in contributing to the growth of total population. This can be explained by this city’s huge demand for investment and qualified labors. Since the 1990s large-scale urban regeneration and foreign investment have triggered a sharp increase in population growth and rapid development of industrial parks as well as economic zones. For example, it was reported that the urban fringe areas such as Pongdong, Baoshan, Jiading, and Minhang attracted 2.67 million people who came to seek investment and employment during 1990–2000 [24]. As a result, in order to accommodate the increasing population and provide more employment opportunities, a lot of new houses and industrial facilities were developed in the urban fringe and suburban satellite towns such as Pudong, Baoshan, Yangpu, Minhang, Jiading, Qinpu, and Songjiang, where both traditional and advanced manufacturing industry enjoyed a boom. Inevitably, the city has expanded extensively and spatially, and therefore the former boundary between suburban satellite towns and the city proper became unclear.

**Figure 4.**
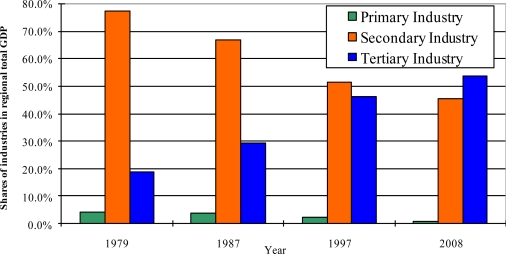
Shares of industries in total GDP during 1979 and 2008.

Shanghai has recently experienced an impacting period of industrial transformation during its way toward prosperous development [18,25]. Figure 4 shows that the shares of agriculture/primary industry (mainly farming, forestry, fishery, husbandry and ranching), secondary industry (mainly mining, refining, manufacturing, energy and water supply, construction) and tertiary industry (mainly service and trade) in total GDP were 3.98%, 77.23%, and 18.79%, respectively, in 1979; however, they were 0.82%, 45.52%, and 53.66%, respectively, in 2008.

Simultaneously, as shown in Table 10, in response to significant changes in sector composition and a sharp decrease in cropland amount, among constituent ratio of total population for major professions, the percentage of population engaging in farming decreased by 13.84%, from 25.02% in 1979 to 11.18% in 2008. On the one hand, advances in techniques and successful practice in intensive agriculture can partly help explain this reason. On the other hand, this indicated the ongoing trends of agricultural land loss and urban growth, which were witnessed with rapid expansion of industrial parks, urban and rural settlements, and infrastructures. The strong competition between urban expansion and agriculture, forestry and other rural land use triggered the conversion of low return lands to high return developmental use, forests and shrubs were cleared for cultivation and more croplands were used to meet increasing demand for urban development [26–27]. As a result, under the growing pressure of land request for accelerated industrialization and urbanization, farmers who lost their lands would have to seek employment or establish their own industries to survive. Therefore, significant socio-economic transformation occurred, and such a situation would lead to new demands for land resources.

### 4.4. Implications for Land Use Policy towards a Sustainable Globalizing Megacity

Due to complicated political and socio-economic factors during the 1950–1980s, many big cities in mainland China such as Beijing, Shanghai, and Tianjin, and so on were transformed into industrial cities, but the demand for urban regeneration and land development were highly constrained with the planned economy system [28]. Take Shanghai for example; there were 5,397 major industries in the city proper in 1985, accounting for 50.6% of total number of industries in Shanghai. The proportion of industrial land use accounted for 24.36% and industrial land use *per capita* was 14.39 m^2^. Thus, proportion of industrial land use and industrial land use *per capita* in Shanghai were both much higher than the other international megacities such as Paris, London, and Chicago [29]. Since the 1990s this city has witnessed its prosperous development in meeting its goals to be an international economic, financial, and trade center to drive the economic growth of the Yangtze River Basin [30]. For example, as part of achieving the targets set for the coming EXPO 2010 in Shanghai, many traditional industrial polluters within the city proper were closed or moved to make way for the development of the central business district (CBD) and urban green space [25]. Besides, with the powerful but successful implementation of government-oriented urban generation policies, old residences in the city proper were demolished and nearly 900,000 residents were resettled in urban fringe and suburban towns. This resulted in significant population agglomeration and enhancement of infrastructures in the urban fringe and suburban towns [24]. However, there is still a long way for Shanghai to be an excellent participant in global competition. During the process of shaping this city as a globalizing metropolis with an excellent investment environment, huge investments were received by the industrial and service sectors. This made it possible to maintain high economic growth and provided numerous employment opportunities, which, in turn, attracted more investments and accelerated urban agglomeration. Thus, it is predicable that contribution of agricultural sector will be negligible and land conversion from cropland to urban development will be accelerated. However, due to very scarce land resource and overloading population in Shanghai, to achieve the economic goals at the loss of cropland, water, and the other lands is not sustainable. Therefore, tidal lands along the coastline were reclaimed and used for animal habitats, cropland, oil refinery, harbor, international airport, warehouse, and the other infrastructures. Over the past decades local big industries such as the Baosteel group, Shanghai Petrochemical group, and Pudong International Airport were established by closing and reclaiming tidal lands. The newly emerging land, which was conversed from tidal land, played an important role in buffering and balancing conflicts between urban development and protection of cropland and some semi-natural patches with ecological importance. For example, at present the Dongtan Wetland Park in Congming County is one of the key habitats for local and regional avian and fishes. Besides, Jiuduan Sa, which is a rapidly expanding siltation-rise isle in the neighborhood of Pudong International Airport, has been the preferred destination for birds [31], and therefore effectively decreased the potential risk of flight accidents since the late 1990s.

On the other hand, with the acceleration of tidal land reclaimation, it is very difficult to reclaim land at the high tidal flats, whereas reclaiming land at the low tidal flats lead to exposure of shallow sea topography and low efficiency in piling up sediment [32]. Besides, as proven by many case studies worldwide, reclaiming land on the high and low tidal flats may cause pronounced degradation in the coastal environment and loss of biological diversity [33–35]. Moreover, in response to dramatic changes in land use and land cover, an ongoing increase in the intensity and extent of urban heat island and regional land subsidence were observed [36–38]. In addition, air pollution, water pollution, water shortage crisis, and flood losses have been more severe than ever. This indicated a very pronounced deterioration in the environment since the 1990s [25,32,39]. Thus, for decision-makers, environmentalists, and farmers who were involved in issues of land use conversion, there has been an urgent to call for action to change the current sprawling patterns of urbanized and urbanizing area. Asian, European and American megacities may provide useful experiences, for example compact city and multi-core pattern are both optional solutions [40–43]. Future land use and urban planning policy aiming at win-win between sustainable land use and economic growth merit a second look.

## 5. Conclusions

In this paper, an integrated approach combining remote sensing and GIS techniques was employed to quantitatively characterize the patterns of land use and cover change (LULC) and further examine the relationship between land use dynamics and driving forces in the fast-growing Shanghai Metropolis, China. Over the study period, urban/built-up land increased by 210.18%, from 585.3 km^2^ in 1979 to 1,815.5 km^2^ in 2008. Total population grew 66.05%, from 11.37 million in 1979 to 18.88 million in 2008. Therefore, rapid increases in both urban/built-up expansion and population led to dramatic changes in land use and land cover, which was witnessed by sharp decreases in croplands, water bodies, and bare lands. On the other hand, due to adverse environmental impacts it was proven that closing and reclaiming land at the tidal flats might not be a practicable way to meet the demand of land use for urban development. Given an excessively dense population, massive resource consumption, and very scarce land resource, these adverse factors have greatly impaired this city’s capacity to meet the challenges presented by international competition and providing a sustainable environment. Thus, rational urban planning policy must be made to decrease the adverse effects of urbanization and enhance the sustainability for this city.

## Figures and Tables

**Table 1.** 1979 accuracy assessment of land use classification (Kappa index and overall accuracy).

	*Urban/Built-up land*	*Cropland*	*Forest and shrub*	*Water*	*Tidal land*	*Bare land*
Urban/Built-up land	30	0	0	1	1	3
Cropland	0	30	9	0	0	0
Forest and shrub	0	17	35	0	0	0
Water	3	0	0	37	5	0
Tidal land	2	1	0	5	32	1
Bare land	6	0	0	0	5	27
PA (%)	77.13					
UA (%)	76.88					
OA (%)	76.40					
Kappa index	0.75					

Note: in this table, PA, UA, and OA represent Producer’s accuracy, User’s accuracy, and overall accuracy, respectively.

**Table 2.** 1987 accuracy assessment of land use classification (Kappa index and overall accuracy).

	*Urban/Built-up land*	*Cropland*	*Forest and shrub*	*Water*	*Tidal land*	*Bare land*
Urban/Built-up land	43	0	0	0	2	5
Cropland	0	39	5	0	0	0
Forest and shrub	0	5	23	0	2	0
Water	0	0	0	28	5	0
Tidal land	3	0	0	8	29	5
Bare land	8	5	0	0	5	30
PA (%)	76.93					
UA (%)	77.18					
OA (%)	76.8					
Kappa index	0.72					

Note: in this table, PA, UA, and OA represent Producer’s accuracy, User’s accuracy, and overall accuracy, respectively.

**Table 3.** Accuracy assessment of land use classification (Kappa index and overall accuracy) for 1997.

	*Urban/Built-up land*	*Cropland*	*Forest and shrub*	*Water*	*Tidal land*	*Bare land*
Urban/Built-up land	44	0	0	0	2	1
Cropland	0	38	0	0	0	0
Forest and shrub	0	9	32	0	0	0
Water	0	0	0	35	0	0
Tidal land	2	1	0	11	20	3
Bare land	10	2	0	0	3	37
PA (%)	83.48					
UA (%)	82.81					
OA (%)	82.4					
Kappa index	0.79					

Note: in this table, PA, UA, and OA represent Producer’s accuracy, User’s accuracy, and overall accuracy, respectively.

**Table 4.** 2008 accuracy assessment of land use classification (Kappa index and overall accuracy).

	*Urban/Built-up land*	*Cropland*	*Forest and shrub*	*Water*	*Tidal land*	*Bare land*
Urban/Built-up land	35	0	0	0	1	5
Cropland	0	33	9	0	0	0
Forest and shrub	0	7	35	0	0	0
Water	0	0	0	37	0	0
Tidal land	2	1	2	5	33	1
Bare land	11	0	0	0	3	30
PA (%)	81.68					
UA (%)	81.74					
OA (%)	81.2					
Kappa index	0.77					

Note: in this table, PA, UA, and OA represent Producer’s accuracy, User’s accuracy, and overall accuracy, respectively.

**Table 5.** Land use transformation matrix in 1979–1987 (unit: km^2^).

Class	Urban/Built-up	Cropland	Forest & shrub	Water	Tidal land	Bare land	sum_1979
Urban/Built-up	214.6	301.2	25.9	12.8	16.1	14.7	585.3
Cropland	358.1	4,252.0	190.0	34.7	62.7	142.6	5,040.1
Forest & shrub	91.3	376.4	33.6	23.0	30.9	26.9	582.1
Water	19.9	58.1	11.4	3,283.4	185.8	17.7	3,576.3
Tidal land	6.7	40.8	9.4	41.3	110.0	11.3	219.5
Bare land	8.2	13.7	1.1	0.7	3.5	0.7	28.0
sum_1987	698.9	5,042.2	271.4	3,395.8	409.0	214.0	10,031.4

Note: The rows and columns contain data of 1979 and 1987 respectively.

**Table 6.** Land use transformation matrix in 1987–1997 (unit: km^2^).

Class	Urban/Built-up	Cropland	Forest & shrub	Water	Tidal land	Bare land	sum_1987
Urban/Built-up	338.3	284.7	11.6	56.3	2.7	5.3	698.9
Cropland	466.9	4,326.2	72.0	151.4	20.7	6.8	5,043.9
Forest & shrub	40.5	201.8	8.7	16.3	3.1	1.0	271.5
Water	22.0	61.2	2.0	3,227.5	61.3	20.2	3,394.3
Tidal land	33.1	161.5	1.3	114.6	77.6	20.6	408.8
Bare land	28.5	161.4	5.9	12.2	4.5	1.4	214.0
sum_1997	929.3	5,196.9	101.6	3,578.3	170.0	55.2	10,031.4

Note: The rows and columns contain data of 1979 and 1987 respectively.

**Table 7.** Land use transformation matrix in 1997–2008 (unit: km^2^).

Class	Urban/Built-up	Cropland	Forest & shrub	Water	Tidal land	Bare land	sum_1997
Urban/Built-up	589.6	184.8	36.3	22.7	0.5	95.4	929.2
Cropland	1,064.0	3,608.8	134.8	123.6	3.2	262.7	5,197.0
Forest & shrub	23.3	57.5	11.5	5.0	0.0	4.3	101.6
Water	98.6	189.9	9.3	3,123.2	80.7	76.9	3,578.5
Tidal land	24.7	34.2	3.0	24.5	39.6	44.1	169.9
Bare land	15.6	12.5	1.3	15.4	3.2	7.2	55.2
sum_2008	1,815.7	4,087.6	196.1	3,314.4	127.2	490.5	10,031.4

Note: The rows and columns contain data of 1979 and 1987 respectively.

**Table 8.** Land use transformation matrix in 1979–2008 (unit: km^2^).

Class	Urban/Built-up	Cropland	Forest & shrub	Water	Tidal land	Bare land	sum_1979
Urban/Built-up	293.5	202.1	22.4	22.7	0.4	44.5	585.5
Cropland	1,222.7	3,303.3	125.3	119.1	0.2	268.9	5,039.5
Forest & shrub	165.6	305.6	31.6	46.4	0.2	32.8	582.2
Water	87.1	167.4	4.2	3077.9	120.6	118.4	3,575.6
Tidal land	39.7	93.2	9.0	46.9	5.7	25.1	219.7
Bare land	6.9	15.5	3.5	1.3	0.8	0.8	28.8
sum_2008	1,815.5	4,087.1	196.1	3,314.3	128.0	490.5	10,031.4

Note: The rows and columns contain data of 1979 and 1987 respectively.

**Table 9.** The single land use dynamic degree of study area in different periods.

*Class*	*1979–1987*	*1987–1997*	*1997–2008*	*1979–2008*
Urban/Built-up	2.43%	4.12%	8.67%	7.25%
Cropland	0.01%	0.38%	−1.94%	−0.65%
Forest&Shrub	−6.67%	−7.82%	8.46%	−2.29%
Water	−0.64%	0.68%	−0.67%	−0.25%
Tidal land	10.78%	−7.30%	−2.25%	−1.44%
Bare land	83.00%	−9.27%	71.66%	56.93%

**Table 10.** Changes of cropland and proportion of population engaged in farming (PPEF) in the study area.

*Year*	*Cropland (km^2^)*	*PPEF (%)*
1979	5,040.14	25.02
1987	5,043.93	18.74
1997	5,196.97	17.72
2008	4,087.13	11.18

## References

[1] Chase T.N., Pielke R.A., Kittel T.G.F., Nemani R.R., Running S.W. (1999). Simulated impacts of historical land cover changes on global climate in northern winter. Clim. Dynam.

[2] Houghton R.A., Hackler J.L., Lawrence K.T. (1999). The U.S. Carbon budget: Contribution from land-use change. Science.

[3] Sala O.E, Chapin F.S., Armesto J.J., Berlow E., Bloomfield J., Dirzo R., Huber-Sanwald E., Huenneke L.F., Jackson R.B., Kinzig A., Leemans R., Lodge D.M., Mooney H.A., Oesterheld M., Poff N.L., Sykes M.T., Walker B.H., Walker M., Wall D.H. (2000). Biodiversity: Global biodiversity scenarios for the year 2100. Science.

[4] Tolba M.K., El-Kholy O.A. (1992). The World Environment 1972–1992: Two Decades of Challenge.

[5] Vitousek P.M. (1994). Beyond global warming: Ecology and global change. Ecology.

[6] Ojima D., Moran E., McConnell W., Smith M.S., Laumann G., Morais J., Young Bill (2005). GLP (2005). Science Plan and Implementation Strategy.

[7] United Nations World Urbanization Prospects: The 2009 Revision Population Database.

[8] Li J.J., Wang X.R., Wang X.J., Ma W.C., Zhang H. (2009). Remote sensing evaluation of urban heat island and its spatial pattern of the Shanghai metropolitan area, China. Ecol. Complex.

[9] Cohen B. (2006). Urbanization in developing countries: Current trends, future projections, and key challenges for sustainability. Technol. Soc.

[10] Chen S., Zeng S., Xie C. (2000). Remote sensing and GIS for urban growth analysis in China. Photogramm. Eng. Remote Sens.

[11] Ozcan H., Cetin M., Diker K. (2003). Monitoring and assessment of land use status by GIS. Environ. Monit. Assess.

[12] Bhatta B. (2009). Analysis of urban growth pattern using remote sensing and GIS: A case study of Kolkata, India. Int. J. Remote. Sens.

[13] Hardin P.J., Jackson M.W., Otterstrom S.M., Jensen RR, Gatrell JD, McLean D (2007). Mapping, measuring, and modeling urban growth. Geo-Spatial Technologies in Urban Environments: Policy, Practice, and Pixels.

[14] Li L., Sato Y., Zhu H. (2003). Simulating spatial urban expansion based on a physical process. Landscape Urban Plan.

[15] Maktav D., Erbek F.S. (2005). Analysis of urban growth using multi-temporal satellite data in Istanbul, Turkey. Int. J. Remote. Sens.

[16] Irwin E.G., Geoghegan J. (2001). Theory, data, methods: developing spatially explicit economic models of land use change. Agr. Ecosyst. Environ.

[17] Cai J., Sit V.F.S. (2003). Measuring world city formation—The case of Shanghai. Ann.Region. Sci.

[18] Shen Z.W. (1994). Development of Pudong: New pattern of urbanization in China. Shanghai Econ. Res.

[19] (2009). Shanghai Municipal Statistics Bureau. Shanghai Statistical Yearbook (2009).

[20] China National Bureau of Statistics (2009). China Statistical Yearbook (2009).

[21] China national committee of agricultural divisions (1984). Technical Regulation of Investigation on Land Use Status.

[22] Jassen I.F.L., Frans J.M., Wel V.D. (1994). Accuracy assessment of satellite derived land-cover data: a review. Photogramm. Eng. Remote Sens.

[23] Chen S.P., Tong Q.X., Guo H.D. (1998). Study on the Mechanism of Remote Sensing Information.

[24] Li J., Nin Y.M. (2007). Population spatial change and urban spatial restructuring in Shanghai since the 1990s. Urban Plan. Forum.

[25] Zhang H., Wang X., Ho H.H., Yong Y. (2008). Eco-health evaluation for the Shanghai metropolitan area during the recent industrial transformation (1990–2003). J. Environ. Manage.

[26] Long H., Tang G., Li X., Heilig G.K. (2007). Socio-economic driving forces of land-use change in Kunshan, the Yangtze River Delta economic area of China. J. Environ. Manage.

[27] Reynolds J.E. (2000). Florida rural land: Competition between agricultural and urban uses. Soil Crop Sci. Soc. FL.

[28] Shen J., Feng Z., Wong K. (2006). Dual-track urbanization in Pearl River Delta. City Plann. Rev.

[29] Yang W.Z., Yin W.H. (2000). Cross-century development of industrial structure in Shanghai. Shanghai Urban Planning Review.

[30] Wu F. (2000). Place promotion in Shanghai, PRC. Cities.

[31] Ge Z.M., Zhou X., Shi W.Y., Wang T.H. (2007). Carrying capacity of shorebirds at Jiuduansha wetland during the migratory seasons. Acta Ecol. Sin.

[32] Li J.F., Dai Z.J., Ying M., Wu R.R., Fu G., Xu H.G. (2007). Analysis on the development and evolution of tidal flats and reclamation of land resource along shore of Shanghai city. J. Nat. Res.

[33] Chen M.R, Han X.F., Liu S.Q. (2000). The effects of reclamation and sustainable development on coastal zone in Shanghai. China Soft Sci.

[34] Goss-Custard J.D., Yates M.G. (1992). Towards predicting the effect of salt-marsh reclamation on feeding bird numbers on the wash. J. Appl. Ecol.

[35] Sato S., Azuma M. (2002). Ecological and paleoecological implications of the rapid increase and decrease of an introduced bivalve Potamocorbula sp. after the construction of a reclamation dike in Isahaya Bay, western Kyushu, Japan. Paleoecology.

[36] Fang Z.L., Wang H.M., Wu J.Z., Lu L.J., Wang Y. (2010). Application research on monitoring land subsidence research in Shanghai using InSAR technology. Shanghai Geol.

[37] Gong S.L., Li C., Yang S.L. (2008). Land subsidence and urban flood prevention safety in Shanghai. Hydro. Eng. Geol.

[38] Liu G., Luo X., Chen Q., Huang D., Ding X. (2008). Detecting land subsidence in Shanghai By PS-networking SAR interferometry. Sensors.

[39] Zhang H., Wang X.R. (2003). Urban land use/cover dynamics of Shanghai metropolitan area and its potential impact on local air environment. Natur. Sci.

[40] Breheny M. (1997). Urban compaction: Feasible and acceptable?. Cities.

[41] Chen H., Jia B., Lau S.S.Y. (2008). Sustainable urban form for Chinese compact cities: Challenges of a rapid urbanized economy. Habitat Int.

[42] Couch C., Karecha J. (2006). Controlling urban sprawl: Some experiences from Liverpool. Cities.

[43] Gusdorf F., Hallegatte S. (2007). Compact or spread-out cities: Urban planning, taxation, and the vulnerability to transportation shocks. Energ. Policy.

